# Activation of M1 macrophages plays a critical role in the initiation of acute lung injury

**DOI:** 10.1042/BSR20171555

**Published:** 2018-04-27

**Authors:** Hui-Lun Lu, Xin-Yan Huang, Yi-Feng Luo, Wei-Ping Tan, Pei-Fen Chen, Yu-Biao Guo

**Affiliations:** 1 Department of Respiratory Medicine, The Second People’s Hospital of Longgang District, Shenzhen, China; 2 The Division of Pulmonary and Critical Care Medicine,The First Affiliated Hospital of Sun Yat-sen University; Institute of Respiratory Diseases of Sun Yat-sen University, Guanzhou, China; 3 The Second Department of Internal Medicine, The Third People’s Hospital of Shenzhen, China

**Keywords:** Acute lung injury, Inflammation, M1 macrohpages, Reactive oxygen species

## Abstract

The goal of the present study was to investigate the role of M1 macrophages in acute lung injury (ALI). To address this, we used lipopolysaccharide (LPS)-treated wild-type and CD11b-DTR mice, and examined their M1 macrophage levels, and the extent of their inflammation and pulmonary injuries. In addition, we evaluated pulmonary function by measuring the expressions of SP-A and SP-B in infiltrated M1 macrophages. Finally, we co-cultured the mouse type II-like alveolar epithelial cells (AT-II) and mouse pulmonary microvascular endothelial cells (PMECs) with M1 macrophages in the presence of TNF-α or H_2_O_2_ and assessed them for viability and apoptosis. After LPS treatment, we observed that the number of pulmonary M1/M2 macrophages and the serum levels of interleukin-1β (IL-1β), tumor necrosis factor α (TNF-α), and reactive oxygen species (ROS) significantly increased. Furthermore, the increase in cytokines was accompanied with the initiation of lung injury indicated by the decreased levels of SP-A and SP-B. In macrophage-depleted CD11b-DTR mice, ALI was attenuated, serum levels of IL-1β, TNF-α and ROS were reduced, and lung levels of monocyte chemoattractant protein-1 (MCP-1) and macrophage inflammatory protein-2 (MIP-2) were decreased. After administering TNF-α and H_2_O_2_, the proapoptotic effect of M1 macrophages on AT-II or PMECs significantly increased, the cell viabilities significantly decreased, and apoptosis significantly increased. Our results suggest that M1 macrophages are recruited to the lungs where they significantly contribute to an increase in TNF-α and ROS production, thus initiating ALI.

## Introduction

Acute lung injury (ALI) is a complex and severe lung disorder marked by respiratory failure, caused by progressive damage of the epithelial and endothelial barriers, flooding of the alveolar compartment, and infiltration of neutrophils into the alveolar space [1]. Acute respiratory distress syndrome (ARDS) is recognized as the most severe form of ALI. Although ARDS was first reported in 1967, both ALI and ARDS continue to be highly prevalent in the United States, because of difficulty in effectively preventing, reducing, and treating them [2]. Sepsis is the leading cause of ALI, resulting in about 74,500 deaths and 3.6 million hospitalized patients each year in the United States alone [2]. However, the underlying mechanisms contributing to the development of ALI are not known.

ALI is characterized by systemic inflammation of the lungs. Recent studies have shown that the infiltration and activation of neutrophils contribute to the pathogenesis of ALI [3,4]. Bacterial infection in ALI affects alveolar macrophages resulting in acute lung inflammation [5]. Furthermore, evidence suggests that macrophages are involved in regulating inflammatory responses and in the resultant lung injury. Interestingly, they are also implicated in the resolution of these responses [6]. Once activated, macrophages produce a variety of inflammatory cytokines such as tumor necrosis factor-α (TNF-α), monocyte chemoattractant protein-1 (MCP-1), macrophage inflammatory protein-2 (MIP-2), and reactive oxygen species (ROS) [7,8]. Even though macrophages play an important role in defending against invading organisms, excessive production of macrophages can contribute to tissue damage.

In the lungs, two different populations of macrophages exist, including alveolar and interstitial macrophages. They are highly versatile cells and their phenotypic and functional properties are influenced by local environmental factors [9]. Based on their pattern of gene expression and roles in host defense, macrophages are classified into classically activated (M1) and alternatively activated (M2) macrophages [10]. In response to the proinflammatory Th1 cytokines, which include TNF-α, interferon-γ (IFN-γ), and lipopolysaccharide (LPS), macrophages undergo M1 activation, characterized by the expression and secretion of proinflammatory factors, such as TNF-α, IL-1β, and inducible nitric oxide synthase (iNOS). On the other hand, the M2 macrophages are induced by the Th2 cytokines that include interleukin-13 and interleukin-4, and are characterized by the expression and secretion of anti-inflammatory factors, such as IL-10 and transforming growth factor-β (TGF-β).

Macrophage levels in lungs increase during lung inflammation, and include both the induction and resolution phases. Some studies suggest that macrophages are associated with the pathogenesis of lung injury [11], while others report that macrophages protect lungs from injury [12]. These conflicting findings can be explained based on either the specific depletion or the recruitment of M1 or M2 macrophages. M1 and M2 macrophages exhibit proinflammatory and anti-inflammatory properties respectively. Therefore, in the present study, we investigated the roles of the different macrophage subpopulations in ALI.

## Materials and methods

### Animals

Eight-to-twelve week old male adult C57BL/6 mice (wild-type) and CD11b-DTR transgenic (Mutant, Mut) mice were obtained from the Nanjing Biomedical Research Institute of Nanjing University. All animal protocols were in accordance with the U.S. National Institutes of Health guidelines and were approved by the Laboratory Animal Care and Use Committee at the The Second People’s Hospital of Longgang District (Shenzhen, China). The mice had free access to food and water and were housed in a specific pathogen-free area with a light/dark cycle of 12 h at a controlled temperature maintained between 20 and 26°C.

### Inoculation of mice with diphtheria toxin

In order to deplete macrophages, the Mut mice were intraperitoneally injected with 4 mg/kg diphtheria toxin (DT. Sigma, St. Louis, MO). To assess the depletion of macrophages, lungs and blood were collected from the animals 1, 7, and 24 days after treatment.

### LPS-induced acute lung injury

The C57BL/6 mice were anesthetized with isoflurane (2%) and intratracheally administered 50 μl of LPS solution (5 mg/kg). The mice were killed by cervical dislocation before LPS treatment and 3, 10, 17, and 24 h after LPS treatment. Subsequently, the lungs and blood were collected for subsequent assays.

### Lung isolation and reperfusion

Mouse lungs were harvested and reperfused, as previously described with some modifications [13]. In brief, the wild-type and Mut mice were anesthetized with intraperitoneal injection of 25% urethane and received tracheostomy followed by continuous ventilation. After injection of heparin through the inferior vena cava, the lungs were exposed and cleared of blood by flushing with 100 ml/kg of Krebs Ringer bicarbonate solution supplemented with 3% fatty acid-free BSA and 10 mM glucose. The lungs were harvested and incubated at 37°C receiving continuous ventilation at 60 cycle/min and perfusion with the blood at 2 ml/min. Blood was collected from both the LPS-treated wild-type and DT-administered Mut mice. After 2 h, the lungs were collected for subsequent assays.

### Histological examination

Seven days after administering DT to the wild-type mice and administering saline or DT to the Mut mice, the animals were treated with LPS. Twenty-four hours later, the mice were killed and the lungs were collected and fixed with 10% formalin, and embedded with paraffin. Next, slices were obtained and stained with hematoxylin and eosin. Finally, microscopic images were obtained by an inverted microscope. Apoptosis was evaluated using the terminal deoxynucleotidyl transferase dUTP nick end labeling (TUNEL) kit (Beyotime, Beijing, China) as per the manufacturer’s protocol.

### Preparation of alveolar macrophages and M1 macrophage stimulation

After anesthesia with 25% urethane, the mice were killed by bloodletting through the femoral artery. Subsequently, the lungs were perfused with heated D-Hanks solution five times. The perfusate was collected and centrifuged at 1500 rpm for 15 min. The pellet was resuspended in 5 ml of PBS and recentrifuged. The pellet was resuspended and cultured in RPMI 1640 with 10% FBS, 100 U/ml penicillin/streptomycin (Invitrogen, Glasgow, U.K.), and 10 ng/ml recombinant GM-CSF (ImmunoTools). The medium was changed every other day for 7 days and M1 macrophages were harvested.

### Cell line and culture

Mouse pulmonary microvascular endothelial cells (PMECs) and mouse type II-like alveolar epithelial cells (AT-II cells) were obtained from American Type Culture Collection. The PMECs were cultured in endothelial cell growth medium MV2 (Promocell, Germany) at 37°C in 5% CO_2_. DMEM containing 2 mM glutamine, 10% FBS, and 100 U/ml penicillin/streptomycin was used as growth medium at 37°C in 5% CO_2_ for incubation of AT-II cells. A transwell two-chamber system for 12-well plates (0.4 μm, Corning Costar) was used and cells were seeded at a density of 2.5 × 10^5^ cells/well in the lower chamber coated with collagen I and cultured for 12 h. Subsequently, M1 macrophages were added to the upper chamber, and the plates were incubated at 37°C in 5% CO_2_. Using either single culture or co-culture with M1 macrophages, PMECs and AT-II cells were treated with TNF-α at 20 ng/ml, H_2_O_2_ at 200 μM, or PBS as control. Finally, the PMECs and AT-II cells were harvested for the viability assay at 24, 48, and 72 h after treatment, as well as for apoptosis and protein expression assays 72 h after treatment.

### Quantitative real-time PCR

At 0, 3, 10, 17, and 24 h after administration of LPS, the wild-type mice were killed by cervical dislocation. Total RNA was isolated from the collected lungs with Trizol (Invitrogen, Grand Island, NY) according to the manufacturer’s protocol. With a Bestar^TM^ qPCR RT kit (Applied Biosystems), 1 μg of total RNA was reverse-transcribed in a 20 μl of PCR reaction. The mRNA levels of IL-1β, iNOS, IL-10, and CD206 were determined by a Real-time PCR (Stratagene Mx3000P) with the primers provided in Table 1. The PCR reaction was conducted at 94°C for 2 min followed by 40 cycles at 94°C for 20 s, 58°C for 20 s, 72°C for 20 s. β-Actin, a housekeeping gene, was used as a control and the relative expressions of the genes were analyzed by the 2^−ΔΔ*C*T^ method.

**Table 1:** Primer pairs used for the RT-PCR

Gene	Forward primers (5′- 3′)	Reverse Primer (5′- 3′)
β-Actin	CATTGCTGACAGGATGCAGA	CTGCTGGAAGGTGGACAGTGA
IL-1β	CAGGCAGTATCACTCATTGT	AGGCTTTTTTGTTGTTCATCTC
iNOS	TCCGAAGCAAACATCACATT	TCCACAACTCGCTCCAAGA
IL-10	AAGGGTTACTTGGGTTGCC	CTCTTATTTTCACAGGGGAGA
CD206	TAGATTTTGTGGCTTGGGC	TGGTGTCGTGGGTGTGGTA

### Western blot

Total protein was extracted from lung tissue homogenates. The protein concentration was determined using the BCA protein assay kits (Beyotime, Shanghai, China). Equal amounts of protein were electrophoresed and then transferred to polyvinylidene difluoride membranes (Bio-Rad, Hercules, CA). After blocking in 5% skim milk for 1 h at room temperature, the membranes were incubated at 4°C overnight with primary antibodies against SP-A, SP-B,MCP-1, MIP-2, CD11c, IL-1β, iNOS, CD206, IL-10, and GAPDH (Santa Cruz Biotechnology). Subsequently, they were washed and then incubated with peroxidase conjugated anti-rabbit or anti-mouse secondary antibodies (Santa Cruz Biotechnolog) for 1 h at room temperature. Finally, the membranes were examined with Lumi-Light ECL substrate (Thermo Fisher Scientific).

### Flow cytometry

The different phenotypes of macrophages were examined using flow cytometric direct immunofluorescence. CD206 was used as a pan-macrophage marker, and iNOS and CD206 were used as specific markers of M1 and M2 macrophages, respectively [14,15]. The cells recovered from the blood were rinsed in PBS and incubated for 30 min on ice with the following specific antibodies (all from eBioscience, San Diego, CA): anti-mouse CD68-FITC, anti-mouse iNOS-PE, anti-mouse CD206-FITC, and the isotype control. After fixing in 2% paraformaldehyde, flow cytometry analysis was performed. Intracellular ROS levels were measured by the Reactive Oxygen Species Assay Kit (Beyotime Biotechnology, China). Briefly, the cells were treated with NAC 2 h before treatment with geridonin and then stained with 10 μM DCHF-DA at 37°C for 30 min. After washing, the cells were assayed by BD LSR II flow cytometry. The PMECs and AT-II cells from each group were detached with 0.25% trypsin, and then were stained with annexin V/propidium iodide (PI) according to the kit manufacturer’s instructions (Beyotime, Beijing, China). The percentages of cells undergoing apoptosis were analyzed by flow cytometry immediately after staining.

### Enzyme-linked immunosorbent assay (ELISA)

The serum levels of IL-1β, TNF-α, IL-10, and TGF-β were quantified by ELISA with the specific Duoset ELISA development system kits (R&D Systems). Briefly, 100 µl of serum and standard samples were added in duplicate into the wells. The plate was covered and incubated overnight at 4°C. After removing the unbound antigens, 100 µl of detection antibody was added. After removing any excess polyclonal antibodies, 100 µl of HRP-conjugated IgG was added. Following a final wash, the bound peroxidase activity was quantified using 100 µl of 3,3′,5,5′-tetramethylbenzidine. After 10 min, the reaction was stopped and OD values at 450 nm were measured using a plate reader (Bio-Rad, NY, U.S.A.). The levels in individual samples were calculated based on standard curves.

### Cell viability assay

After treatment, the viability of PMECs and AT-II cells were analyzed using the Cell-Counting Kit-8 (CCK-8) proliferation assay kit as per the manufacturer’s instructions. The absorption intensity was determined at 450 nm by a microplate spectrophotometer (TECAN, Australia).

### Hoechst staining

Cell apoptosis was determined by nuclear staining with Hoechst 33342 (Beyotime, Beijing, China) according to a previous protocol. Briefly, after treatment, the cells were fixed with 4% formaldehyde and permeabilized with 0.5% Triton X-100. Subsequently, Hoechst 33342 was added to the medium at a concentration of 5 μg/ml. Images were captured with a fluorescent microscope (Olympus, Japan).

### Statistical analysis

All data are expressed as mean ± SD. Statistical analysis was performed using two-tailed Student’s *t* test by GraphPad Prism. *P*<0.05 was considered significant. Representative results of 2–3 independent experiments with each sample in duplicates or triplicates are shown.

## Results

### LPS treatment increased the number of M1/M2 macrophages in lungs

**Figure 1:**
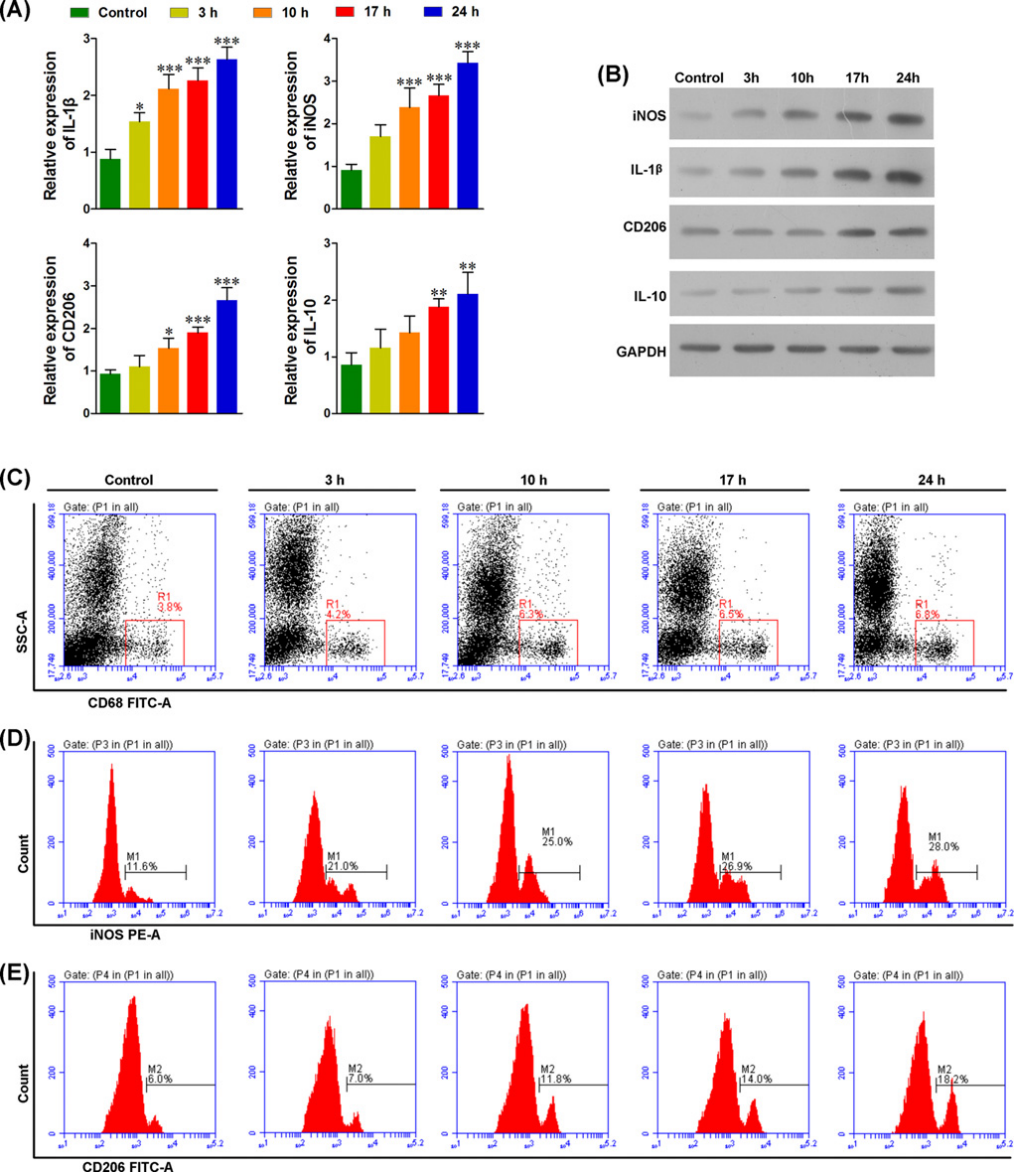
M1 and M2 macrophages are significantly induced after LPS treatment (**A**) Real-time PCR analysis showed that the RNA levels of IL-1β and iNOS (markers of M1 macrophages), and IL-10 and CD206 (markers of M2 macrophages) were significantly increased in a time-dependent manner after LPS treatment. (**B**) Western blot analysis showed that the protein levels of IL-1β, iNOS, IL-10, and CD206 were significantly increased in a time-dependent manner. (**C**) The CD68+ inflammatory cells were significantly increased in the lungs after LPS treatment. (**D**) The M1 macrophages (iNOS+) were significantly increased within 3–24 h after LPS treatment. (**E**) The M2 macrophages (CD206+) were significantly increased within 10 h to 24 h after LPS treatment; *P<0.05, **P<0.01, ***P<0.001 compared with each pretreatment, *n* = 5.

We treated mice with LPS to evaluate its effects on the M1/M2 macrophage profile. Western blots and RT-PCR were used to determine M1/M2 populations in the lungs with the markers for IL-1β and iNOS for M1 macrophages, and markers for CD206 and IL-10 for M2 macrophages. As shown in Figure 1A,B, LPS treatment significantly increased IL-1β, iNOS, CD206, and IL-10 at both the transcriptional and translational levels, indicating that LPS treatment significantly increased the number of M1/M2 macrophages in the lungs. We noted that IL-1β and iNOS levels increased 3 h after LPS treatment. This was earlier than the CD206 and IL-10 levels, which increased 10 h after LPS treatment. These findings suggest that LPS treatment induced the M1 population earlier compared with the M2 population. Consistent with these data, flow cytometric analysis showed a significant increase in the proportion of macrophages (Figure 1C), and also showed an earlier increase in number of M1 than M2 macrophages (Figure 1D,E). The varied profiles suggest that M1 macrophages play an important role in the ALI.

### Macrophage polarization increased the levels of ROS and proinflammatory cytokines

**Figure 2:**
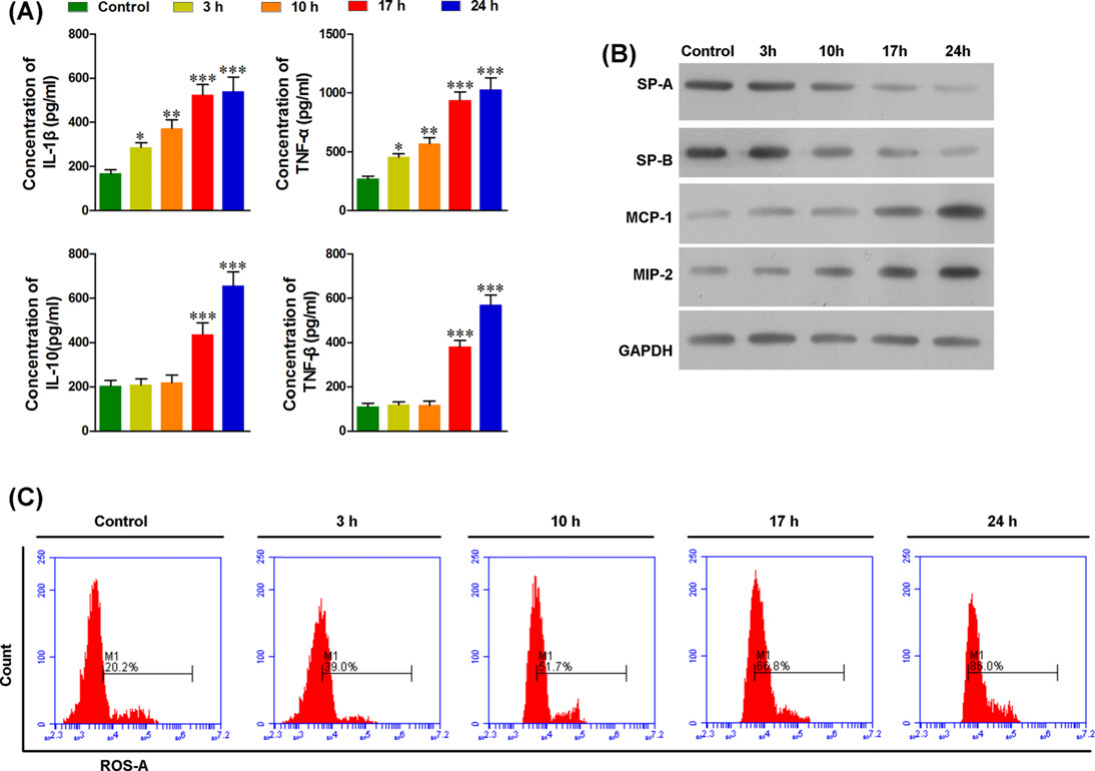
LPS treatment increased the inflammatory cytokines and ROS in the blood and induced acute lung injury (**A**) The serum levels of IL-1β and TNF-α were significantly increased at the onset of 3 h after LPS treatment, and serum levels of IL-10 and TNF-β were significantly increased at the onset of 17 h after LPS treatment. (**B**) The levels of SP-A and SP-B in the lungs were significantly decreased at the onset of 10 h after LPS treatment and in a time-dependent manner, whereas the levels of MCP-1 and MIP-2 in the lungs were significantly increased in a time-dependent manner. (**C**) The serum levels of ROS before LPS treatment and at 3, 10, 17, and 24 h after LPS treatment were determined with flow cytometry. ROS levels increased at the onset of 3 h after LPS treatment and in a time-dependent manner; *P<0.05, **P<0.01, ***P<0.001 compared with each pretreatment, *n* = 5.

After LPS treatment, blood was collected to determine the activity of M1/M2 macrophages. As shown in Figure 2, levels of IL-1β, TNF-α, IL-10, TGF-β, and ROS in the serum were significantly increased after LPS treatment. We found an initial increase in IL-1β, TNF-α, and ROS levels 3 h post LPS treatment, followed by a sustained increase. For IL-10 and TGF-β, the initial increase was observed 17 h after LPS treatment (Figure 2A,C). These data suggest that the increased levels of IL-1β, TNF-α, and ROS result from M1 macrophages and not from M2 macrophages. SP-A and SP-B are markers of lung function. A decrease in protein levels of these markers was observed at the onset of 10 h after LPS treatment (Figure 2B), indicating lung injury was initiated between 3 and 10 h after LPS treatment. Together with the pulmonary levels of M1/M2 macrophages and the expression of the inflammatory cytokines, M1 macrophages might play an important role in ALI rather than M2, and IL-1β, TNF-α, and ROS might contribute to ALI. As the important inflammatory cytokines involved with the inflammation response, pulmonary MCP-1 and MIP-2 were also significantly increased 3 h after LPS treatment, indicating that peripheral macrophages are recruited to the lungs before ALI initiation.

### Macrophage depletion attenuated ALI

**Figure 3:**
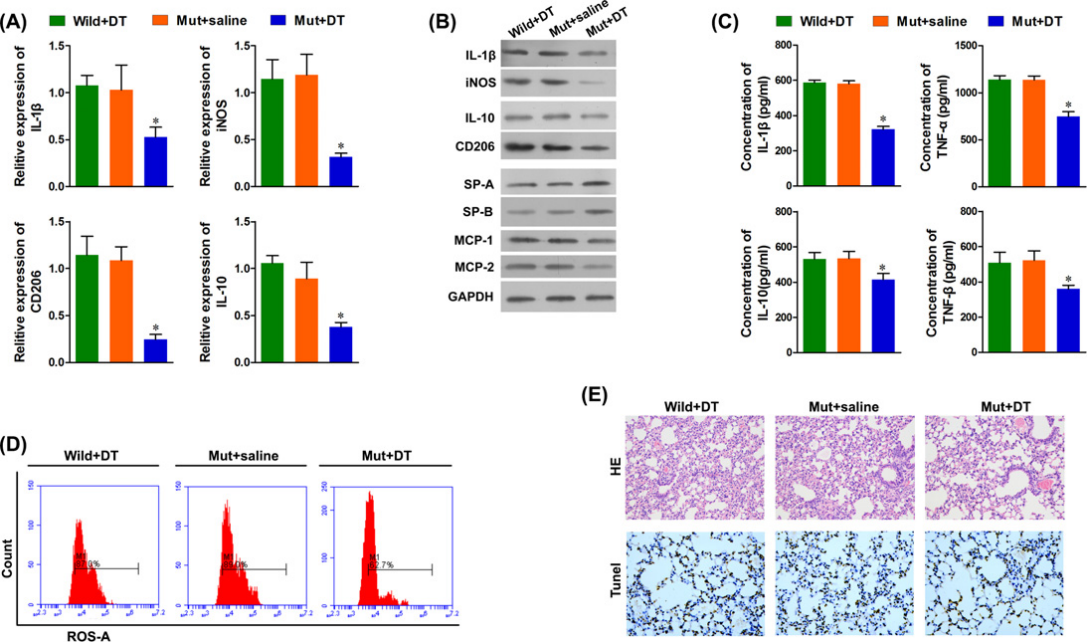
Number of macrophages in the lungs of CD11b-DTR mice was significantly decreased after DT treatment (**A**) mRNA levels of M1 and M2 macrophage markers were detected with real-time PCR. (**B**) Protein levels of M1 and M2 macrophage markers were detected with Western blots, and the protein levels of SP-A and SP-B in the lungs were significantly increased, the protein expressions of MCP-1 and MIP-2 were significantly decreased in the lungs of the Mut + DT mice. (**C**) The serum levels of IL-1β, TNF-α, IL-10 and TNF-β were significantly decreased in the LPS-treated macrophage depleted Mut mice. (**D**) The serum levels of ROS were significantly decreased in the LPS-treated macrophage depleted Mut mice. (**E**) H&E staining (magnification ×400) and TUNEL staining of representative lung sections (magnification ×400). *P<0.05 compared with each pretreatment, *n* = 5.

Next, we observed the effect of diphtheria on the M1 and M2 macrophage populations in mutant (Mut, CD11b-DTR) mice. As shown in Figure 3A–F, we observed that the mRNA and protein levels of CD11b, IL-1β, iNOS, CD206, and IL-10 in mice lungs were significantly decreased at 1, 7, and 14 days after DT treatment (Figure 3A,B). However, the mRNA levels on day 14 were higher than those on day 7, indicating that the number of pulmonary macrophages might gradually increase 7 days after DT treatment. Furthermore, in Mut mice treated with DT, the serum levels of IL-1β, TNF-α, IL-10, and TNF-β were significantly decreased, as compared with both wild-type mice treated with DT and Mut mice treated with saline (Figure 3C). In addition, flow cytometry revealed decreased levels of ROS in Mut mice treated with DT (Figure 3D). Furthermore, septal edema was observed 24 h after LPS treatment in both the wild-type mice treated with DT and the Mut mice treated with saline, and this was not observed in the Mut mice treated with DT. Similarly, a significant increase in apoptosis of epithelial cells was observed in control mice compared with Mut mice treated with DT (Figure 3E). These data indicate that M1 macrophages contribute to ALI.

### Peripheral macrophages are recruited to the lungs

**Figure 4:**
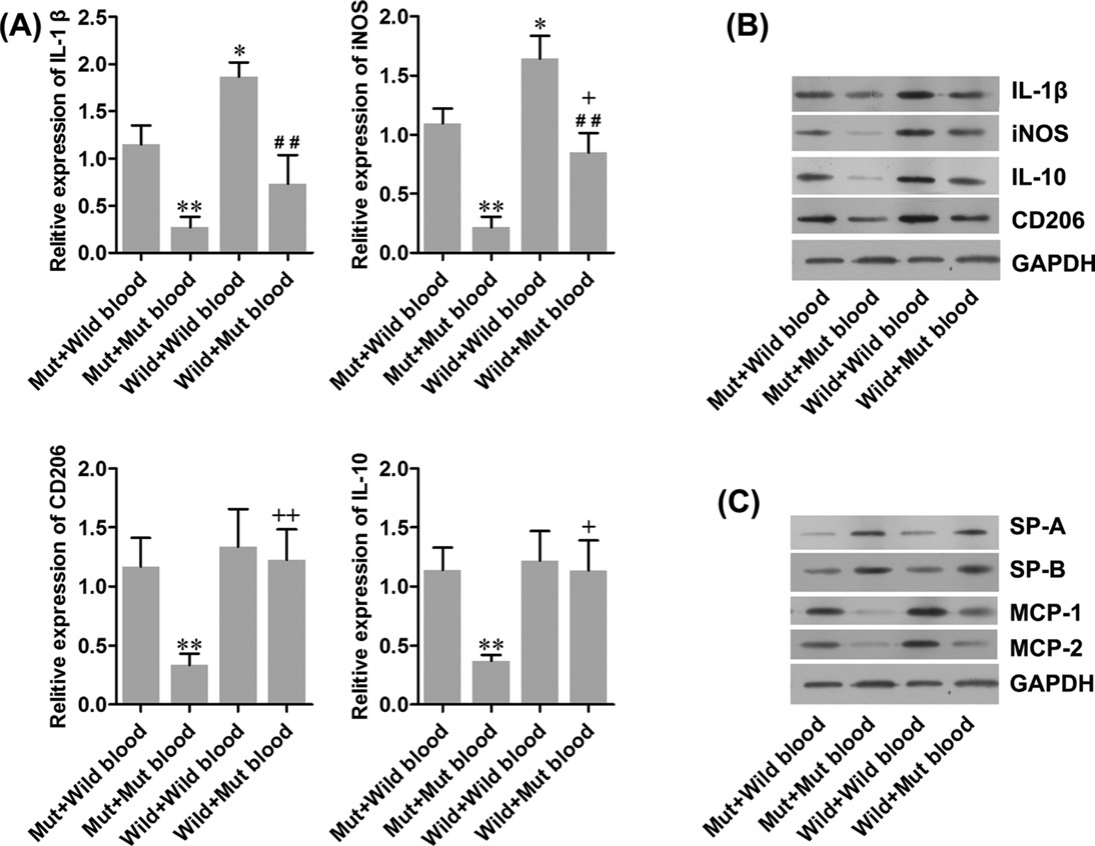
Effect of peripheral macrophages on pulmonary damage Blood from mice with ALI were collected and perfused to isolated wild-type or Mut mice lungs. (**A**) mRNA levels of M1 and M2 macrophages markers were detected with real-time PCR. (**B** and **C**) Protein levels of M1 and M2 macrophages markers, as well as the protein levels of SP-A, SP-B, MCP-1, and MCP-2 were detected with Western blot. GAPDH was used as an internal control protein and the same samples were used in panels (B) and (C). *P<0.05, **P<0.01 compared with Mut plus wild-type blood; +*P*<0.05, ++*P*<0.01 compared with Mut plus Mut blood; ##*P*<0.01 compared with wild-type plus wild-type blood. Wild-type: normal C57BL/6 mice. Mut: DT treated CD11b-DTR mice.

Since our data suggested that M1 macrophages contribute to ALI, we investigated the infiltration of macrophages to the lungs. We collected the blood from mice with ALI and perfused the blood to isolated wild-type and Mut mice lungs. As shown in Figure 4A, perfusing blood from mice with ALI into wild-type mice significantly increased the expressions of IL-1β and iNOS in the wild-type lungs compared with the Mut lungs. We did not observe any change in expression of CD206 and IL-10. Moreover, in isolated Mut mice lungs, perfusion with Mut blood decreased the expression of four genes compared with that of wild-type blood. The changes in protein levels of these four genes were further validated by Western blotting (Figure 4B). The data suggested that in wild mice, M1 macrophages and not M2 macrophages are significantly increased due to recruitment from the blood to the lungs. We also noted that MCP-1 and MIP-2 levels were significantly increased, and SP-A and SP-B levels were significantly decreased in the lungs after perfusion with the blood from the wild mice with ALI. The increase or decrease occurred in the wild-type and Mut lungs, which is consistent with the observations in M1 macrophages (Figure 4C). This indicated MCP-1 and MIP-2 might play an important role in the recruitment of M1 macrophages to the lungs and thus, contribute to lung dysfunction.

### M1 macrophages promote TNF-α- and H_2_O_2_-induced epithelial and endothelial cell apoptosis

**Figure 5:**
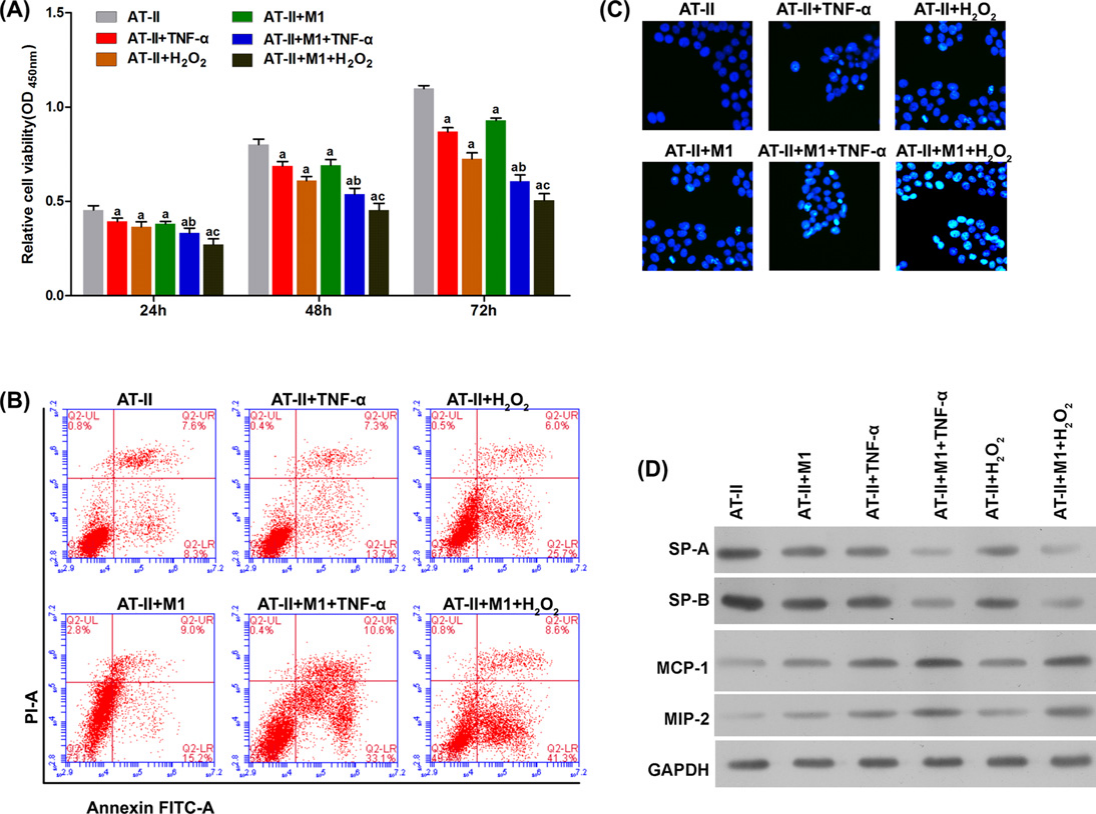
M1 macrophages promote apoptosis and dysfunction in AT-II cells in the presence of TNF-α and H_2_O_2_ (**A**) TNF-α and H_2_O_2_ significantly decreased the viability of AT-II cells in a time-dependent manner. By flow cytometry (**B**) and Hoechst staining (**C**), the apoptotic effect on AT-II cells by TNF-α and H_2_O_2_ was significantly enhanced by M1 macrophages. By Western blot (**D**), the pulmonary function indicated by SP-A and SP-B was significantly impaired by TNF-α and H_2_O_2_, and this was significantly facilitated by M1 macrophages. M1 macrophages increased protein levels of MCP-1 and MIP-2. ^a^*P*<0.05 compared with single culture of AT-II without treatment (AT-II group); ^b^*P*<0.05 compared with co-culture system without TNF-α treatment (AT-II + TNF-α group); ^c^*P*<0.05 compared with co-culture system without H_2_O_2_ treatment (AT-II + H_2_O_2_ group).

**Figure 6:**
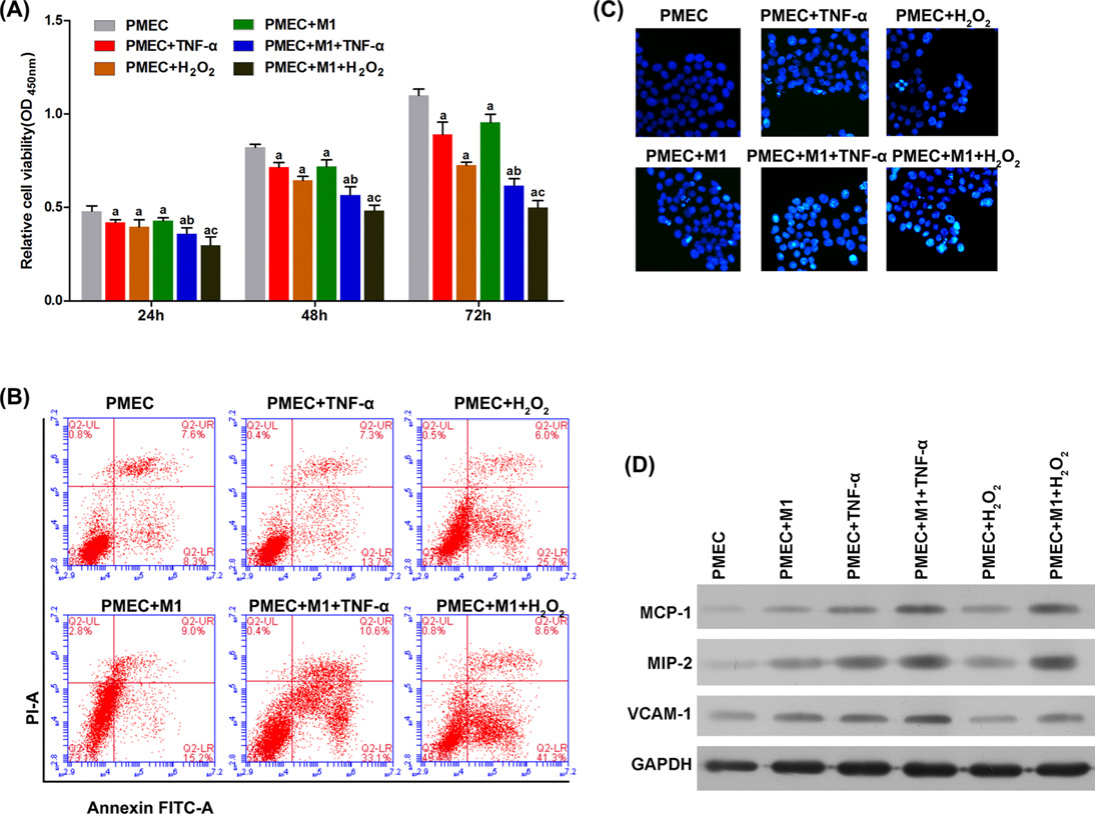
M1 macrophages promote apoptosis and dysfunction in PMECs in the presence of TNF-α and H_2_O_2_ (**A**) TNF-α and H_2_O_2_ significantly decreased the viability of PMECs in a time-dependent manner, and this was amplified by M1 macrophages. By flow cytometry (**B**) and Hoechst staining (**C**), the apoptotic effect on PMECs by TNF-α and H_2_O_2_ was significantly promoted by M1 macrophages. By Western blot (**D**), the protein levels of MCP-1 and MIP-2 in PMECs were significantly induced by TNF-α and H_2_O_2_, and this was significantly facilitated by M1 macrophages. ^a^*P*<0.05 compared with single culture of PMECs without treatment (PMECs group); ^b^*P*<0.05 compared with co-culture system without TNF-α treatment (PMECs + TNF-α group); ^c^*P*<0.05 compared with co-culture system without H_2_O_2_ treatment (PMECs + H_2_O_2_ group).

To evaluate the role of M1 macrophages in pulmonary epithelial and endothelial cells during ALI, we co-cultured AT-II cells or PMECs with M1 macrophages *in vitro*. As shown in Figures 5A and 6A, co-culture with M1 macrophages, and stimulation with TNF-α- or H_2_O_2_-induced significant apoptosis in AT-II cells and PMECs 24, 48, and 72 h after treatment. This was further validated with flow cytometry and Hoechst staining (Figures 5B,C and 6B,C). Furthermore, the apoptotic effect of M1 macrophages was further enhanced by stimulating with TNF-α or H_2_O_2_. M1 macrophage-induced injury to epithelial cells was confirmed by evaluating the expressions of SP-A and SP-B (Figure 5D). The injury to endothelial cells was reflected in changes to MCP-1 and MIP-2 protein levels (Figure 6D).

## Discussion

ALI has a significant impact on public health and patient outcomes [1]. Since pulmonary inflammation contributes to the initiation and development of ALI, substantial efforts have been made to understand the pathological and restorative role of inflammatory factors contributing to ALI. As a critical component of the inflammatory system, macrophages are important effectors and regulators of tissue homeostasis in both normal and lesioned lungs. M1 and M2 macrophages are the major subsets of macrophages that exhibit proinflammatory and anti-inflammatory effects respectively. In the present study, we confirmed that M1 macrophages play a critical role in the pathogenic initiation of ALI in CD11b-DTR mice [16,17]. Furthermore, we demonstrated that this was mediated by TNF-α and H_2_O_2_.

Inflammation plays an important role in the pathogenesis of ALI. As the first line of defense against organisms inhaled into the respiratory tract, macrophages are recruited to the lungs as part of the early pathological response. In our study, we found that in macrophage-depleted Mut mice, lung injuries were significantly attenuated compared with the injuries observed in Mut and wild-type mice. This suggested that macrophages critically contribute to ALI. In addition, we observed that the number of M1 macrophages significantly increased after 3 h post LPS-treatment. This is probably due to direct induction by LPS [18,19]. The early pathological stage of ALI comprises acute inflammation with monocyte and neutrophil infiltration in the alveolar space, which critically contributes to ALI [20]. Alveolar macrophages also play an important role in the migration of neutrophils to the lungs. With increased stimulation of foreign antigens, macrophages are activated to the M1 subtype, which secretes proinflammatory cytokines, like inflammatory monocyte recruitment (MCP-1) and neutrophil recruitment (MIP-2), and helps exacerbate and sustain inflammatory responses [21]. Consistent with this, we observed a significant increase in the expressions of MIP-2 and MCP-1 3 h after LPS treatment. Furthermore, we observed that the expressions of MCP-1 and MIP-2 lung endothelial and epithelial cells were significantly increased by activated M1 macrophages. We observed an attenuation in alveolar function 10 h after LPS treatment. These findings suggest that macrophages differentiate into the M1 subtype right after LPS treatment and consequently recruit monocytes and neutrophils to the lungs. We thus speculate that M1 macrophages plays an important role in the initiation and maintenance of ALI by inducing inflammatory cell migration. On the other hand, the levels of M2 macrophages significantly increased 10 h after LPS treatment. Furthermore, the contribution of M1 macrophages to ALI was demonstrated by the co-culture test. Based on their anti-inflammatory properties and activation time, we hypothesize that M2 macrophages might play an important role in the resolution of inflammation and recovery of lung injury [6].

Proinflammatory cytokines contribute to acute injury in organs, including lungs, kidneys, and livers [22]. In the present study, we observed that the plasma levels of the proinflammatory cytokines, TNF-α, and IL-1β, were significantly increased 3 h post LPS treatment. Since M1 macrophages are an important source of TNF-α and IL-1β, M1 macrophages secreting TNF-α and IL-1β might contribute to ALI. Several studies have indicated that TNF-α plays a central role in the development of ALI, by acting locally to stimulate chemotaxis, recruit, and activate neutrophils [23–27]. In addition, TNF-α induces apoptosis in alveolar epithelial lung microvascular endothelial cells [28,29]. IL-1β is one of the most biologically active cytokines in the early phase of ALI and has been shown to contribute to ALI by inducing neutrophil recruitment and activation, and increasing vascular permeability via the integrin pathway [30].

Oxidative injury to the lungs mediated by ROS is another facet of ALI. ROS can lead to cell injury by various mechanisms, including altering protein activity and enhancing the expression of proinflammatory cytokines [31–34]. Neutrophils, macrophages, and monocytes are examples of some sources of ROS that occur during ALI [35]. In our study, we observed that macrophage depletion significantly decreased the levels of ROS, implicating macrophages in both producing ROS during ALI, and contributing to ALI. Since M1 macrophages facilitate initial and sustained ALI, we infer that M1 macrophages induce ALI by ROS.

In conclusion, our results suggest that M1 macrophages function as a critical contributor of ALI. Furthermore, this involvement is mediated through TNF-α and ROS. However, the present study lacks clinical research and still need further research to demonstrate the role of M2 macrophages. More cytological and molecular findings may provide the theoretical basis for new methods of clinical treatment to ALI.

## Abbreviations

ALI: acute lung injury
ARDS: acute respiratory distress syndrome
IL-1β: interleukin-1β
iNOS: inducible nitric oxide synthase
LPS: lipopolysaccharide
PMEC: pulmonary microvascular endothelial cell
ROS: reactive oxygen species
TNF-α: tumor necrosis factor-α
